# Association Between Myasthenia Gravis and Systemic Lupus Erythematosus as a Comorbid State

**DOI:** 10.7759/cureus.14719

**Published:** 2021-04-27

**Authors:** Moeez Ali, Mohamed Riad, Prakash Adhikari, Sanket Bhattarai, Ashish Gupta, Eiman Ali, Jihan A Mostafa

**Affiliations:** 1 Internal Medicine, California Institute of Behavioral Neurosciences & Psychology, Fairfield, USA; 2 Internal Medicine, Piedmont Athens Regional Medical Center, Athens, USA; 3 Research, California Institute of Behavioral Neurosciences & Psychology, Fairfield, USA; 4 Psychiatry, California Institute of Behavioral Neurosciences & Psychology, Fairfield, USA

**Keywords:** systemic lupus erythematosus, thymectomy, sle, myasthenia gravis, autoimmune disease, acetylcholine receptor antibody, anti-nuclear antibody

## Abstract

Systemic lupus erythematosus (SLE) and myasthenia gravis (MG) are autoimmune states which have presentational similitude. Both conditions test serologically positive for anti-nuclear antibodies and require exceptional differential diagnostic acumen to segregate one from the other. The hypothesized factors provoking these diseases may be immunological, genetic, hormonal, or environmental and can be better understood by large-scale controlled epidemiological studies. Biochemical factors such as variation in CXC (an α chemokine subfamily), CXCL13, and granulocyte-macrophage colony-stimulating factor levels are assumed to play a pivotal role in the pathogenesis of SLE and MG; however, further studies are required to understand their exact mechanism and effect on the underlying autoimmune diseases. Following this, another precipitating factor for this overlap is believed to be thymectomy which is performed to eliminate MG symptoms. Although thymectomy is the effective treatment modality in MG patients, other findings and data support the view that this procedure may lead to the development of other autoimmune states such as SLE. It is evident from previously published data and case reports that patients with one autoimmune disease who underwent thymectomy contracted SLE and became more susceptible to other autoimmune diseases compared to the general population. Post-thymectomy follow-up of patients provides us with mechanistic clues for understanding the development of SLE-MG overlap; hence, in MG patients who have undergone thymectomy, any clinical and immune serological SLE suspicion should be carefully evaluated.

## Introduction and background

Systemic lupus erythematosus (SLE) is an autoimmune disease with no bias toward any organ and is defined by the presence of anti­-dsDNA and anti-­SM antibodies, which may contribute to the occurrence of sustained pro-inflammatory state in the body [1,2]. Autoimmune diseases and disorders associated with direct proliferation and differentiation of B-cells (polyclonal activation of B-lymphocytes) play a significant role in the pathogenesis of SLE. The presentation of signs and symptoms related to several other autoimmune diseases are ubiquitous and can concur with previously diagnosed SLE [3-5]. According to a study, the aforementioned case was observed in 30% of SLE patients [3-5]. In fact, observations have shown presentation of Sjögren’s syndrome, rheumatoid arthritis, thrombocytopenia, anti-phospholipid syndrome, and hypothyroidism as comorbid states with SLE [3]. Myasthenia gravis (MG) is a chronic, organ-specific autoimmune disease in which elements of the motor end plate are affected by the immune system, specifically by the anti-R-acetylcholine (Ach) autoantibodies and T-lymphocytes directed against Ach receptors, specific tyrosine kinase receptors (MuSK), and muscle proteins [6-8]. This autoimmune state has an express inclination toward cranial muscles and may vary in severity [9,10]. SLE and MG are similar in a way that both of the autoimmune states may present with thymus hyperplasia in addition to being serologically positive for anti-nuclear antibodies (ANA) [11,12]. Interestingly, different studies over the years have suggested highly variable statistical data for the occurrence of this rare overlapping condition; for example, a study conducted over a period of 7.5 years involving 380 SLE patients concluded an incidence of MG in 0.25% of the subjects in contrast to a lower incidence of 0.02% in the general population [13,14]. Not only in adults but these conditions may also exist in juveniles as juvenile myasthenia gravis (JMG) and juvenile-onset SLE with annual prevalence of 0.9-2.0 per million and 0.37-0.90 per 100,000, respectively. According to reports, the possibility of simultaneous occurrence of these conditions is very rare (approximately 9.5 per 1,012) [15,16]. Regarding management, an interventional approach for MG is thymectomy as the thymus is assumed to provoke autoantibody production; however, this methodology has a negligible effect on pre-existing SLE and may precipitate other autoimmune diseases [17].

This review article intends to highlight the association between MG and SLE as co-morbid states, as well as their synergistic effects on the patients. In addition, the role of thymectomy, risk factors, and the clinical approach to these disorders will also be analyzed. This article will also try to identify the gaps, limitations, and variations of the data available for this rare overlapping case by featuring case studies as there have been no impactful, controlled epidemiological studies on this subject.

## Review

Myasthenia gravis and polyautoimmunity

Autoimmune diseases are a heterogeneous group of immune states in which the body’s own organs are marked and targeted as a result of immune intolerance to self-antigens [18]. Due to environmental, hormonal, genetic, and several immunological variances, patients with one autoimmune disease are prone to develop additional autoimmune diseases. Some studies report as many as 80/100,000 new autoimmune disease occurrences per year, and the frequency is more prevalent in women than in men [18-22].

The most common autoimmune disease association with MG is thyroid disease with an incidence of 5-10%, whereas the incidence of MG in patients with previously diagnosed thyroid disease is only 0.2% [23]. In addition, rheumatoid arthritis, SLE, and pernicious anemia are also some common findings in association with MG [23]. In a study carried out among six individuals with associated autoimmune diseases and MG, AChR antibody tests presented with four positive results, out of which two were non-thymectomized with Grave’s disease and two were thymectomized with hypothyroidism (Hashimoto’s thyroiditis), microthymoma B1B2, and thymic hyperplasia. In addition, the remaining two patients with hyperthyroidism (Grave’s disease) and rheumatoid arthritis were negative for the anti-AChRa test which shows that the coexistence of multiple autoimmune disorders is not uncommon [24].

MG is becoming an increasingly prevalent autoimmune disease, more frequently in women than in men. According to studies, its presentation with other autoimmune disorders as a co-morbid state is drawing attention to the possible common basis for their coexistence and their impact on the severity, management, and prognosis of the disease.

Systemic lupus erythematosus and myasthenia gravis overlap

SLE and MG are diseases that share certain similarities, especially with a high incidence rate in women. As mentioned earlier, the patients predisposed to an autoimmune disease are more prone to develop another autoimmune disease. According to studies, this could be between 13% and 22% for patients with MG [25,26]. According to a Swedish investigation carried out among 2,045 MG cases, positivity for other autoimmune diseases was seen in 449 (22%) patients. The most common findings were rheumatoid arthritis, hypothyroidism, type 1 diabetes, and psoriasis. In comparison to a control group of the general population, the patients with established MG had higher incidence of developing other autoimmune diseases [25,27]. On the contrary, another engaging study carried out by Canadian researchers over a span of nine years including 380 subjects concluded in only one positive diagnosis of MG [13]. One of the plausible justification for the contradiction between the aforementioned studies could be misdiagnosing muscle incapacitation as a consequence of glucocorticoid therapy (glucocorticoid myopathy) rather than a symptom of MG [27].

Another study was carried out among 13 patients with MG and preexisting SLE, out of which 11 had satisfactory data available. The patients were diagnosed according to the criteria set by the American College of Rheumatology (ACR) and are summarized in Table 1 [13].

**Table 1:** Common clinical characterizations in SLE and MG overlap as noted in 11 out of 13 patients. *anti-DNA or anti-Smith, anti-cardiolipin antibodies, or lupus anticoagulant ACR: American College of Rheumatology; SLE: systemic lupus erythematosus; MG: myasthenia gravis

Total number	11
ACR criteria for SLE diagnosis, n (%)	
Malar rash	2 (18.2%)
Oral ulcers	2 (18.2%)
Photosensitivity	2 (18.2%)
Renal disorder	2 (18.2%)
Discoid rash	3 (27.3%)
Neurological disorder	4 (36.4%)
Serositis	6 (54.5%)
Hematological disorder	8 (72.7%)
Arthritis	10 (90.9%)
Immunological disorder*	10 (90.9%)
Anti-nuclear antibody	11 (100%)

Interestingly, majority of the subjects included in the study were relatively young and had a mean SLE duration of eight years when they were diagnosed with MG. Out of these 13 patients, most were non-white with only one Caucasian (7.7%), followed by three Black (23.1%), one Hispanic (7.7%), four Asian (30.8%), and four patients of unknown race/ethnicity (30.8%).

Patients with SLE, with or without neurological findings, should be referred for anti-AChR testing followed by an electromyography examination in order to confirm or exclude MG.

Immunological factors and pathophysiology

An alpha chemokine subfamily (CXC) has been emphasized as a major factor in the pathogenesis of both states [28,29]. The reason noted in studies shows that these chemokines are responsible for mobility of several immunoreactive cells via chemoattraction. They may even mediate the activation of dendritic cells, monocytes, T, B, and NK cells, basophils, and eosinophils, and are also involved in angiogenesis [30]. CXCL13 is believed to precipitate SLE in patients with established MG due to its interaction with B and T-lymphocytes as proven by studies on animal models [28].

Furthermore, another common factor between the two diseases is granulocyte-macrophage colony-stimulating factor (GM-CSF) which can be found exogenously and endogenously. It is interesting to note that in addition to vascular endothelial cells, fibroblasts, mast cells, monocytes and macrophages, and T and B cells are also responsible for the endogenous production of GM-CSF, which represents its noteworthy immunological association. On the other hand, exogenous GM-CSF may be used as a treatment approach to synthesize bone marrow-derived granulocytes and macrophages [31]. Although GM-CSF administration has shown a reduction in neutrophil apoptosis, which presents as a possible SLE treatment option, further research and sophisticated studies are required to understand its role in the underlying autoimmune diseases.

Thymectomy: treatment or a risk factor for autoimmune diseases?

Thymus is a lymphoid organ present in the mediastinum that maintains immune competence and plays a vital role in the regulation of cell-mediated immunity by secretion of regulatory hormones and controlling T-cell differentiation into helper (CD4) and killer/cytotoxic (CD8) lymphocytes [32-34]. Thymic involvement has been observed in up to 75% of myasthenia patients, of which about 15% have an underlying thymoma and the rest have thymic hyperplasia [35]. Thymectomy is a well-known practice for prospective management of thymoma-associated autoimmune diseases such as pure red cell aplasia and MG, and has proven to be a good prognostic tool for symptomatic management of underlying autoimmune diseases [33]. Given the premise, surprisingly, there have been several cases in which thymectomy has resulted in exacerbation of the underlying autoimmune diseases in addition to contracting additional ones, specifically SLE [33].

Pathological anomalies (like hyperplasia or thymoma) in the thymus may result in cell dysfunction, which leads to a rise in the number of CD4+ T-lymphocytes, which is a notable pathogenic factor in MG [36]. Under normal homeostatic conditions of the body, T-lymphocytes act by inhibiting CD4+ lymphocytes in order to prevent autoantibody production; however, the interaction of these cells with B-lymphocytes results in an opposite response and triggers an organ damaging autoimmune response [37]. In addition, it has been hypothesized that CD4+ and CD25+ lymphocyte deficiency is not only involved in connective tissue pathogenesis but also predisposes to SLE and MG [37,38].

It is doubtful that post-thymectomy SLE occurs by a coincidence in MG patients, which is evident by different published case reports on this subject. For example, a case series of four patients with SLE and MG overlap studied their response to different treatment strategies prior to and post-thymectomy. The data are summarized in Table 2 [38].

**Table 2:** A case series of four patients who underwent thymectomy and other treatment strategies for SLE and MG overlap management. M: male; F: female; MG: myasthenia gravis; SLE: systemic lupus erythematosus; APS: anti-phospholipid syndrome

Variables/Findings	Case 1	Case 2	Case 3	Case 4
Age/Gender	56/F	57/M	58/F	62/F
Age at MG onset	10	54	58 (initially diagnosed as SLE)	33
SLE	Present	Present	Present	Present
Thymectomy	Yes	No	No	Yes
Treatment (initial)	Thymectomy, pyridostigmine	Cholinesterase inhibitor	Hydroxychloroquine (discontinued by personal decision)	Thymectomy, pyridostigmine
Treatment (later)	Hydroxychloroquine	Mycophenolate mofetil, hydroxychloroquine	None	Hydroxychloroquine
Other pathological findings/abnormalities	-	Synovitis, history of pulmonary embolism	History of seizures and mini stroke, APS	Right facial hemiparesis

Based on these findings, we can observe that the subjects who underwent thymectomy to alleviate MG symptoms ended up with complete remission of the disease; however, a consequential result was SLE development which draws our attention to the risks associated with thymectomy and the need to follow-up on patients who underwent the procedure.

## Conclusions

The objective of this review article is to shed light on updated information regarding SLE and MG overlap for researchers and readers. The mutual association of SLE and MG deserves more than a casual mention. Although rare, both conditions have similar presentations, and it is established that their prevalence is higher in women than in men. Not only in adults but rarely these morbid states can be found in juveniles as JMG and juvenile systemic lupus erythematosus, and it is interesting to note that one disease may precede another. Given this premise, the analogy between different autoimmune diseases shows that individuals with one autoimmune disease are prone to develop another compared to the general population.

Further inspection and monitoring of subjects presenting with increased levels of CXCL13 and GM-CSF and with SLE and MG demonstrate their active role in pathogenesis of both diseases; however, further research can shed more light on the precise mechanism of these biochemical changes and their synergistic effect on autoimmune states. In addition, thymectomy is hypothesized to be responsible for the emergence of novel autoimmune diseases, including the subject under study. Not only this but conditions like idiopathic portal hypertension, Hashimoto’s disease, cutaneous vasculitis, and anti-phospholipid syndrome have been observed in post-thymectomy state. It is hypothesized that thymectomy may stimulate excessive autoantibody production in patients predisposed to SLE; however, the exact mechanism remains unclear, which leads us to the conclusion that epidemiological studies on a larger scale are required to understand the pathophysiological changes in patients. Furthermore, exploration of new treatment strategies and better diagnostic skills and methods will improve the patient prognosis.
